# High-Intensity Static Stretching in Quadriceps Is Affected More by Its Intensity Than Its Duration

**DOI:** 10.3389/fphys.2021.709655

**Published:** 2021-07-05

**Authors:** Kosuke Takeuchi, Shigeru Sato, Ryosuke Kiyono, Kaoru Yahata, Yuta Murakami, Futaba Sanuki, Riku Yoshida, Masatoshi Nakamura

**Affiliations:** ^1^Department of Physical Therapy, Graduate School of Medicine, Kobe University, Kobe, Japan; ^2^Institute for Human Movement and Medical Sciences, Niigata University of Health and Welfare, Niigata, Japan; ^3^Department of Physical Therapy, Niigata University of Health and Welfare, Niigata, Japan

**Keywords:** short duration, shear elastic modulus, range of motion, rectus femoris, stretch tolerance

## Abstract

A previous study reported that 3-min of high-intensity static stretching at an intensity of 120% of range of motion (ROM) did not change the muscle stiffness of the rectus femoris, because of the overly high stress of the stretching. The purpose of this study was to examine the effects of high-intensity static stretching of a shorter duration or lower intensity on the flexibility of the rectus femoris than that of the previous study. Two experiments were conducted (Experiment 1 and 2). In Experiment 1, eleven healthy men underwent static stretching at the intensity of 120% of ROM for two different durations (1 and 3 min). In Experiment 2, fifteen healthy men underwent 3-min of static stretching at the intensity of 110% of ROM. The shear elastic modulus of the quadriceps were measured. In Experiment 1, ROM increased in both interventions (*p* < 0.01), but the shear elastic modulus of the rectus femoris was not changed. In Experiment 2, ROM significantly increased (*p* < 0.01), and the shear elastic modulus of the rectus femoris significantly decreased (*p* < 0.05). It was suggested that the stretching intensity (110%) is more important than stretching duration to decrease the muscle stiffness of the rectus femoris.

## Introduction

Static stretching is used to improve flexibility of muscles and to prevent injuries (Takeuchi et al., 2019). Flexibility is often measured by using the range of motion (ROM). A change in ROM after static stretching is attributed to changes in stretching tolerance and the stiffness of the muscle (Behm et al., 2016). Stretching tolerance is evaluated by measuring the passive torque during passive joint movement (Behm et al., 2016) or pain during stretching (Nakamura et al., 2021). The stiffness of the muscles can be measured by using an isokinetic dynamometer machine and shear wave elastography and is denoted as the muscle-tendon unit stiffness (Takeuchi and Nakamura, 2020b,c) and muscle stiffness (Nakamura et al., 2021), respectively.

Muscle strain is a frequent sports injury and occurs most often in hamstrings, quadriceps [especially the rectus femoris (RF)], and gastrocnemius (Schuermans et al., 2016; Soligard et al., 2017; Orchard et al., 2020). To prevent muscle strain, it is important to decrease the muscle-tendon unit stiffness (Watsford et al., 2010; Pickering Rodriguez et al., 2017). Static stretching acutely decreases the muscle-tendon unit stiffness of hamstrings (Magnusson et al., 1996; Matsuo et al., 2013; Cabido et al., 2014), rectus femoris (Caliskan et al., 2019), and gastrocnemius (Mizuno et al., 2013; Konrad et al., 2019). Therefore, a recent review study recommends using static stretching as a fundamental warm-up component before recreational sport participation due to its potential positive effect on flexibility and musculotendinous injury prevention (Chaabene et al., 2019).

Recently, the acute effects of high-intensity static stretching on the muscle-tendon unit stiffness have been investigated. The intensity of static stretching is determined according to the ROM and point of discomfort (POD) of each participant. Previous studies performed high-intensity static stretching at the intensity of 120% ROM and 120% POD and showed that high-intensity static stretching effectively decreased the muscle-tendon unit stiffness of the hamstrings (Kataura et al., 2017; Takeuchi and Nakamura, 2020b,c) and the muscle stiffness of the medial gastrocnemius (Fukaya et al., 2020). On the other hand, Nakamura et al. (2021) compared 3-min of three different stretching intensities (80, 100, and 120% ROM) on the muscle stiffness of the quadriceps and showed that the muscle stiffness of the RF decreased only after static stretching at the intensity of 100% ROM. Moreover, Nakamura et al. (2021) reported that static stretching at the intensity of 120% ROM could put excessive stress on the quadriceps, which hampers the effects of the high-intensity stretching. Therefore, the effects of high-intensity static stretching may differ depending on the targeted muscles. To develop an effective static stretching method to prevent the muscle strain of the RF, it is necessary to examine the conditions of high-intensity static stretching that are effective in decreasing the muscle stiffness of the RF.

The stress of static stretching increases with stretching duration and intensity. Santos et al. (2020) reported that the muscle-tendon unit stiffness of the hamstrings did not change after 3-min of high-intensity static stretching at the intensity of the numerical rating scale (NRS) Levels 9–10 (an NRS of Level 10 indicates the worst possible discomfort). On the other hand, Kataura et al. (2017) showed that 3-min of static stretching at the intensity of 120% ROM (NRS Level 5) effectively decreased the muscle-tendon unit stiffness of the hamstrings. These studies indicated that the muscle-tendon unit stiffness of the hamstrings does not change if the stretching intensity is excessive. However, Takeuchi and Nakamura (2020b, c) reported that 20-s of high-intensity static stretching at the intensity of NRS Level 9 effectively decreased the muscle-tendon unit stiffness of the hamstrings. Taken together, these results indicated that a shorter duration of high-intensity static stretching can decrease the muscle-tendon unit stiffness of the hamstrings even if the stretching intensity was excessively high. It was hypothesized that the muscle stiffness of the rectus femoris, as well as that of the hamstrings, may not change when the intensity or duration of high-intensity static stretching is excessive. The purpose of the present study was to examine the effects of high-intensity static stretching of a shorter duration or lower intensity on ROM and muscle stiffness of the RF, vastus lateralis (VL), and vastus medialis (VM) than that of the previous study (Nakamura et al., 2021), and to show the optimal high-intensity static stretching for decreasing the muscle stiffness of the RF.

## Materials and Methods

### Experimental Design

**FIGURE 1:**
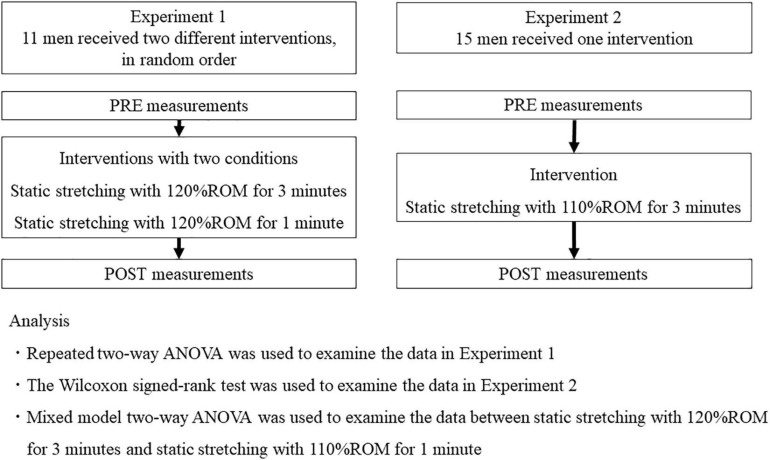
Experimental design. ROM, range of motion; ANOVA, analysis of variance.

Two experiments were conducted (Experiment 1 and 2) to examine the effects of high-intensity static stretching of shorter durations or lower intensities than that of the previous study (intensity of 120% ROM and duration of 3 min) (Nakamura et al., 2021; Figure 1). For Experiment 1, a randomized repeated measure experimental design was used to compare two different durations (1 min vs. 3 min) of static stretching at the intensity of 120% ROM on knee flexion ROM, muscle stiffness and stretching pain of the quadriceps. The participants visited two times, that is, once a day on two separate days, with an interval of >72 h, and received two interventions, in random order. For Experiment 2, the effects of 3-min of static stretching at the intensity of 110% ROM on knee flexion ROM, muscle stiffness and stretching pain of the quadriceps were examined. Knee flexion ROM and the shear elastic modulus (RF, VL, and VM) of quadriceps in the dominant leg (ball kicking preference) were measured before and immediately after each intervention. In addition, the visual analog scale (VAS) was used to examine the quadriceps muscle pain magnitude during each stretching session.

### Participants

Eleven (23.8 ± 3.4 years, 168.7 ± 5.4 cm, 62.4 ± 5.5 kg) and fifteen healthy men (23.1 ± 2.9 years, 168.4 ± 6.1 cm, 62.8 ± 6.9 kg) were recruited for Experiment 1 and 2, respectively. All participants did not regularly perform any flexibility or strength training. Participants who had a neuromuscular disease or lower extremity musculoskeletal injury history were excluded. During the experimental period, all participants were instructed not to perform resistance or flexibility training of the lower limbs. Previous studies that examined the effects of high-intensity static stretching reported large effect sizes for the muscle stiffness (Takeuchi and Nakamura, 2020b,c; Nakamura et al., 2021). Therefore, the sample size of the muscle stiffness was calculated with a power of 80%, alpha error of 0.05, and effect size of 0.40 (Experiment 1) and 0.80 (Experiment 2) using G^∗^Power 3.1 software (Heinrich Heine University Düsseldorf, Düsseldorf, Germany), and the results showed that the requisite number of participants for this study was 8 and 15 for Experiment 1 and 2, respectively. There was no significant difference in baseline ROM and the muscle stiffness of the RF between the participants in Experiment 1 and 2 (*p* = 0.22 and 0.52 for an unpaired *t*-test, respectively). All participants were informed of the requirements and risks associated with their involvement in this study and signed a written informed consent document. The study was performed in accordance with the Declaration of Helsinki (1964)^1^. The study was approved by the Ethics Committee of the Niigata University of Health and Welfare, Niigata, Japan, (Procedure #17677).

### Assessment of Knee Flexion ROM

Knee flexion ROM was measured in the same fashion as a previous study (Nakamura et al., 2021). In detail, participants were positioned with a 90° flexion of the hip and knee joint of the non-dominant leg and 30° extension of the hip joint of the dominant leg, as the reference limb position. Afterward, the knee joint was passively and slowly flexed by the investigator from the reference limb position to the knee flexion angle just before the participants started to feel discomfort or pain, not the feeling of quadriceps extension (Akagi and Takahashi, 2013; Sato et al., 2020; Nakamura et al., 2021). The knee flexion ROM was measured using a goniometer twice and the average value was used for further analysis. The reliability of knee flexion ROM was acceptable in this study (intraclass correlation coefficient of 0.930).

### Assessment of the Muscle Stiffness of the Quadriceps Muscle

In this study, the shear elastic modulus was measured to examine any changes in the muscle stiffness of the RF, VL, and VM by using ultrasonic shear-wave elastography (Aixplorer Supersonic Imagine, Aix-en-Provence, France) with a SL10-2 linear probe. The participants were lying on a treatment bed in the neutral hip joint position with a 90° flexed hip and knee joint. The shear elastic moduli of the RF, VL, and VM were measured at the midpoint, 60 and 80% distal between the anterior superior iliac spine and the proximal end of the patella, respectively (Nakamura et al., 2021). The size of the region of interest was 10 × 20 mm^2^ and set near each muscle center, with an analysis area of a 5-mm-diameter circle at the center of the stiffer region (Saeki et al., 2019). Long-axis elastographic images were obtained twice. Based on previous studies (Hirata et al., 2016; Nakamura et al., 2020, 2021), the shear elastic modulus was calculated by dividing the obtained Young’s modulus by three. The average shear elastic modulus value obtained from the duplicate elastographic images was used for analysis. In our previous study, high intraclass correlation coefficients demonstrated the reliability of the shear elastic modulus procedure used in this study (RF of 0.876, VL of 0.909, and VM of 0.960) (Nakamura et al., 2021).

### Assessment of Stretching Pain

The pain of the quadriceps was assessed during each stretching intervention using a VAS with a 100-mm continuous line with “not sore at all” on one side (0 mm) and “very, very sore” on the other (100 mm). Stretching pain assessments were performed during each stretching intervention, three times.

### Static Stretching

The static stretching intervention was performed in a similar fashion to the knee flexion ROM assessment. The intensities of static stretching were calculated based on the knee flexion ROM in the PRE value in each intervention (Kataura et al., 2017; Takeuchi and Nakamura, 2020b,c; Nakamura et al., 2021). At 110 and 120% intensity for Experiment 1 and 2, the angle of static stretching was set to 1.1 and 1.2 times the knee flexion ROM at the PRE value, respectively. For Experiment 1, static stretching at the intensity of 120% ROM was performed for 1 min (three 20-s stretching with 30-s intervals) and 3 min (three 60-s stretching with 30-s intervals). For Experiment 2, static stretching at the intensity of 110% ROM for 3 min (three 60-s stretching with 30-s intervals) was performed. Participants were instructed to be relaxed and raise their torso upright during the stretching intervention.

### Statistical Analyses

The Shapiro–Wilk test was used to test for normality of the PRE-value in Experiment 1 and 2. For Experiment 1, a two-way repeated analysis of variance (ANOVA) was used to examine the effects of time (PRE vs. POST) and intervention (1 min vs. 3 min) on ROM and the shear elastic modulus. For VAS, a two-way repeated ANOVA was used to examine the effects of time (Set 1 vs. Set 2 vs. Set 3) and intervention (1 min vs. 3 min). If a significance was detected, *post hoc* analyses using Bonferroni’s test were performed. For Experiment 2, the paired *t*-test and Wilcoxon signed-rank test were used to compare the PRE and POST values of knee flexion ROM and muscle stiffness (RF, VL, and VM), respectively. For VAS in Experiment 2, a one-way repeated ANOVA was used, and if a significance was detected, *post hoc* analyses using Bonferroni’s test were performed. Moreover, a mixed model ANOVA was conducted to examine the effects of time (PRE vs. POST) and intervention (static stretching with 120% ROM intensity for 3 min vs. static stretching with 110% ROM intensity for 3 min) on ROM and the shear elastic modulus. For VAS, a mixed model ANOVA was used to examine the effects of time (Set 1 vs. Set 2 vs. Set 3) and intervention (static stretching with 120% ROM intensity for 3 min vs. static stretching with 110% ROM intensity for 3 min). Partial eta squared (small = 0.01, medium = 0.06, and large = 0.14) and *r*-values (small = 0.10, medium = 0.30, and large = 0.50) were reported to reflect the magnitude of the differences among each intervention (Cohen, 1988; Cabido et al., 2014; Takeuchi and Nakamura, 2020b). The analyses were performed using SPSS version 25 (SPSS, Inc., Chicago, IL, United States). Differences were considered statistically significant at an alpha level of *p* < 0.05.

## Results

### Experiment 1

#### Knee Flexion ROM

There was no significant two-way interaction (*p* = 0.18, partial eta squared = 0.09), and no main effect for intervention (*p* = 0.08, partial eta squared = 0.15), however, there was a significant main effect for time (*p* < 0.01, partial eta squared = 0.84). Knee flexion ROM significantly increased in both interventions (average ± standard deviation: 1 min, PRE = 128.2 ± 9.2 degrees, POST = 145.9 ± 6.5 degrees; 3 min, PRE = 123.4 ± 11.4 degrees, POST = 136.8 ± 9.8 degrees) (both *p* < 0.01).

#### The Shear Elastic Modulus

There were no significant two-way interactions in the shear elastic modulus of the RF, VL, and VM (*p* = 0.12, partial eta squared = 0.12; *p* = 0.07, partial eta squared = 0.18; *p* = 0.11, partial eta squared = 0.12, respectively), and no main effect for the intervention (*p* = 0.95, partial eta squared = 0.00; *p* = 0.46, partial eta squared = 0.03; *p* = 0.33, partial eta squared = 0.05, respectively) and time (*p* = 0.11, partial eta squared = 0.12; *p* = 0.10, partial eta squared = 0.13; *p* = 0.99, partial eta squared = 0.00, respectively) (Table 1).

**TABLE 1:** Changes in the shear elastic modulus (Experiment 1).

	**Intervention**	**PRE**	**POST**	**% Change**
RF (kPa)	1 min	11.5 ± 1.7	10.9 ± 2.5	94.7 ± 14.1
	3 min	12.1 ± 1.7	12.8 ± 2.1	106.1 ± 14.5
VL (kPa)	1 min	6.0 ± 0.8	5.4 ± 1.0	90.8 ± 8.9
	3 min	6.0 ± 1.3	6.1 ± 1.1	102.1 ± 11.5
VM (kPa)	1 min	7.4 ± 1.6	6.9 ± 2.0	93.8 ± 16.4
	3 min	7.7 ± 1.5	8.2 ± 2.0	106.2 ± 16.5

*Average ± standard deviation. RF, rectus femoris; VL, vastus lateralis; VM, vastus medialis; kPa, kilopascal.*

#### VAS

There was no significant two-way interaction (*p* = 0.42, partial eta squared = 0.04), and no main effect for intervention (*p* = 0.70, partial eta squared = 0.01), however, there was a significant main effect for time (*p* < 0.01, partial eta squared = 0.34) (Table 2). In both interventions, *post hoc* analyses revealed that the VAS value of the Set 3 was significantly smaller than that of the Set 1 (*p* < 0.01) and Set 2 (*p* < 0.05).

**TABLE 2:** Changes in the visual analog scale (VAS) values during the stretching intervention.

**Intervention**	**Set 1**	**Set 2**	**Set 3**
Experiment 1			
1 min	64.0 ± 18.6	53.0 ± 18.3	47.3 ± 16.6*^,†^
3 min	62.0 ± 15.7	57.5 ± 15.9	52.4 ± 16.3*^,†^
Experiment 2	43.2 ± 16.2	36.1 ± 12.7^$^	27.3 ± 14.5*^,†^

Average ± standard deviation. *p < 0.01 (First session vs. Third session). ^†^P < 0.05 (Second session vs. Third session). ^$^P < 0.05 (First session vs. Second session).

### Experiment 2

A paired *t*-test showed that knee flexion ROM significantly increased after the stretching (average ± standard deviation: PRE = 128.7 ± 9.8 degrees, POST = 142.4 ± 7.8 degrees, *p* < 0.01, *r* = 0.86). A Wilcoxon signed-rank test revealed that the muscle stiffness of the shear elastic modulus of the RF significantly decreased (*p* = 0.03, *r* = 0.56), but that of the VL (*p* = 0.16, *r* = 0.36) and VM (*p* = 0.32, *r* = 0.26) were not changed (Table 3). One-way repeated ANOVA revealed a significant effect for the VAS value (*p* < 0.01, partial eta squared = 0.52). *Post hoc* analysis revealed that the VAS value of the Set 2 (*p* = 0.04) and Set 3 (*p* < 0.01) were significantly smaller than that of the Set 1 (Table 2).

**TABLE 3:** Changes in the shear elastic modulus (Experiment 2).

	**PRE**	**POST**	**% Change**
RF (kPa)	12.7 (10.8–14.5)	11.6 (10.5–13.1)*	89.5 ± 18.9
VL (kPa)	6.0 (5.6–6.7)	5.9 (5.5–6.4)	94.5 ± 15.2
VM (kPa)	7.0 (6.4–7.9)	7.1 (6.3–7.8)	97.7 ± 14.8

Median (25–75%). *p < 0.05 (PRE vs. POST). RF, rectus femoris; VL, vastus lateralis; VM, vastus medialis; kPa, kilopascal.

### Comparison Between 120% ROM and 110% ROM Intensity

For knee extension ROM, there was no significant two-way interaction (*p* = 0.46, partial eta squared = 0.04), nor main effect for intervention (*p* = 0.20, partial eta squared = 0.07), however, there was a significant main effect for time (*p* < 0.01, partial eta squared = 0.68). Knee flexion ROM significantly increased in both interventions (both *p* < 0.01).

For the shear elastic modulus of the RF, there was a significant two-way interaction (*p* = 0.03, partial eta squared = 0.19). The shear elastic modulus of the RF significantly decreased only in 110% intensity (*p* = 0.02), but not 120% intensity (*p* = 0.36).

For the shear elastic modulus of the VL and VM, there were no significant two-way interactions (*p* = 0.21, partial eta squared = 0.07; *p* = 0.11, partial eta squared = 0.10, respectively), nor main effect for intervention (*p* = 0.53, partial eta squared = 0.48; *p* = 0.21, partial eta squared = 0.06, respectively) or time (*p* = 0.27, partial eta squared = 0.05; *p* = 0.72, partial eta squared = 0.01, respectively).

For the VAS value, there was no significant two-way interaction (*p* = 0.34, partial eta squared = 0.43), but there was a significant main effect for time (*p* < 0.01, partial eta squared = 0.43) and intervention (*p* < 0.01, partial eta squared = 0.92). The VAS value in 120% ROM intensity was significantly greater than that of 110% ROM intensity (*p* < 0.01). The VAS value of Set 3 was significantly smaller than that of Set 1 (*p* < 0.01) and Set 2 (*p* < 0.01).

## Discussion

In Experiment 1, the result of this study revealed that knee flexion ROM significantly increased after static stretching at the intensity of 120% ROM regardless of stretching durations (1 and 3 min). Takeuchi and Nakamura (2020c) examined the effects of three different durations (10, 15, and 20 s) of high-intensity static stretching in the hamstrings and reported that ROM significantly increased regardless of stretching durations. In addition, our results in Experiment 2 showed that knee flexion ROM significantly increased after static stretching at the intensity of 110% ROM, which is consistent with the previous study (Nakamura et al., 2021). Previous studies suggested that a mechanism of the increase in knee flexion ROM could be involved with a change in stretching tolerance, which is the sensation of pain during passive joint movement (Magnusson, 1998; Weppler and Magnusson, 2010; Behm et al., 2016; Freitas et al., 2018). In Experiment 1 of the present study, the stretching pain was measured by using the VAS value and it decreased gradually in both stretching interventions, which was consistent with the previous study (Nakamura et al., 2021). Moreover, the VAS value was also decreased gradually in Experiment 2. In the present study, all three sets of static stretching were performed at the same knee angle, but the sensation of the pain during the stretching decreased with each stretching session. Therefore, it was possible that changes in stretching tolerance could contribute to changes in ROM in all stretching interventions, although the present study did not measure the value of passive torque at terminal ROM as the index of stretching tolerance (Brusco et al., 2019; Takeuchi and Nakamura, 2020a).

In Experiment 1, the shear elastic modulus of the RF, VL, and VM was not changed after either the 1- or 3-min of static stretching at the intensity of 120% ROM. Takeuchi and Nakamura (2020b) reported that high-intensity static stretching significantly decreased the muscle-tendon unit stiffness of the hamstrings regardless of the stretching durations. It is suggested that the duration of high-intensity static stretching would not have a crucial effect on the changes in muscle stiffness of the quadriceps and muscle-tendon unit stiffness of the hamstrings. On the other hand, in Experiment 2, the shear elastic modulus of the RF significantly decreased after 3-min of static stretching at the intensity of 110% ROM but no significant change in the shear elastic modulus of the VL and VM was shown. Nakamura et al. (2021) showed that the shear elastic modulus of the RF significantly decreased only after static stretching at the intensity of 100% ROM, with no significant changes in the shear elastic modulus of the RF after static stretching at the intensity of 80 and 120% ROM. On the other hand, previous studies showed that static stretching at the intensity of 120% ROM, or more, effectively decreased the muscle-tendon unit stiffness of the hamstrings (Kataura et al., 2017; Takeuchi and Nakamura, 2020b,c) and the muscle stiffness of the medial gastrocnemius (Fukaya et al., 2020). It was suggested that the effects of high-intensity static stretching on the muscle stiffness differs depending on the target muscles, and static stretching at the intensity of 110% ROM or less, rather than at 80% ROM could decrease the shear elastic modulus of the RF.

In the present study, VAS values of static stretching at the intensity of 120 and 110% ROM were approximately 60 and 43.2, respectively. The previous study reported that the VAS values of static stretching at the intensity of 120% and 100% ROM were 59.5 and 13.5, respectively (Nakamura et al., 2021). These data indicated that stretching pain during the static stretching at the intensity of 120% ROM was very high. Nakamura et al. (2021) pointed out that 3-min of static stretching at the intensity of 120% ROM puts excessive stress on the quadriceps, and as a result, the effects of the high-intensity static stretching on muscle stiffness may be hampered by an inflammatory response (Apostolopoulos et al., 2018) and sympathetic nerve activity (Shiro et al., 2012). A strong positive correlation has been reported between the intensity of static stretching and stretching pain (Takeuchi and Nakamura, 2020b,c). Taken together, it is suggested that VAS values of approximately 40 or less are effective when performing high-intensity static stretching for the purpose of decrement in the muscle stiffness of the RF.

There were some limitations in the present study. The participants were not athletes. Therefore, it is necessary to examine the effects of high-intensity static stretching in the quadriceps of athletes who regularly engage in flexibility training, including static stretching. In addition, the intensity of static stretching was defined with reference to ROM (Kataura et al., 2017; Fukaya et al., 2020; Sato et al., 2020; Takeuchi and Nakamura, 2020b). However, since ROM is affected by the subjective factor of stretching tolerance, it was unclear as to the objective intensity of the static stretching. It is necessary to measure passive torque during high-intensity static stretching to examine the objective intensity of the static stretching in detail.

## Conclusion

The effects of different duration (1 and 3 min) of static stretching at the intensity of 120% ROM was investigated (Experiment 1). The results showed that ROM increased in both stretching interventions, but the shear elastic modulus of the RF, VL, and VM showed no change. In Experiment 2, the effects of 3-min of static stretching at the intensity of 110% ROM were examined, and an increment in ROM and decrement in the shear elastic modulus of the RF were found. It is suggested that the intensity of static stretching (110% ROM) is more important than its duration to decrease muscle stiffness of the RF.

## Data Availability Statement

The raw data supporting the conclusions of this article will be made available by the authors, without undue reservation.

## Ethics Statement

The studies involving human participants were reviewed and approved by the Niigata University of Health and Welfare. The patients/participants provided their written informed consent to participate in this study.

## Author Contributions

All authors listed have made a substantial, direct and intellectual contribution to the work, and approved it for publication.

## Conflict of Interest

The authors declare that the research was conducted in the absence of any commercial or financial relationships that could be construed as a potential conflict of interest.

## Footnotes

1 World Medical Association Declaration of Helsinki: Ethical Principles for Medical Research Involving Human Subjects. JAMA 284(23): 3043–3045.
